# Retinal artery occlusion by left atrial myxoma misdiagnosed as thrombus

**DOI:** 10.1186/s40064-016-1990-2

**Published:** 2016-03-22

**Authors:** Jeong-Ho Kim, Ho-Joong Youn, Mi-Hyang Jung, Chang-Yul Oh, So-Hyun Ahn, Woo-Hyun Cho, Jong-Hun Lee, Yong-Seok Lee, Hyo Jin Hyun

**Affiliations:** Division of Cardiology, Department of Internal Medicine, Pohang St. Mary’s Hospital, Pohang, Republic of Korea; Department of Ophthalmology, Pohang St. Mary’s Hospital, Pohang, Republic of Korea; Cardiovascular Center, College of Medicine, Seoul St. Mary’s Hospital, The Catholic University of Korea, Banpo-daero 222, Seocho-gu, Seoul, 137-701 Republic of Korea

**Keywords:** Retinal artery occlusion, Cardiac myxoma, Thrombus, Visual loss

## Abstract

**Introduction:**

Left atrial mass has been known to have a benign course.

**Case description:**

A 55-year-old Asian woman visited to our hospital because of right sided hemiparesis and sudden vision loss in her left eye. She diagnosed as acute cerebral infarction and central retinal artery occlusion by several studies. We detected a large mass in the left atrium and thought this mass as thrombus causing multiple emboli. But her neurologic symptom was aggrevated during therapy and coronary computed tomography angiogram suggested a left atrial myxoma, not thrombus. She underwent the resection of the myxoma.

**Discussion and evaluation:**

Sometimes it could be fatal because it could be source of systemic embolization. Moreover, when the mass is located at unusual site, it is difficult to differentiate thrombus and other benign mass.

**Conclusions:**

We report a patient with a left atrial mass initially presented as multiple embolic infarction. Our case will let ophthalmologists know about the possibility of retinal artery occlusion by cardiac myxoma.

## Introduction

Left atrial myxoma, the most common primary cardiac tumor, is usually fatal unless surgically resected (Fintan O’Rourke et al. 2003). The classic echocardiographic features of a left atrial myxoma include a mobile smooth mass attached to the interatrial septum by a stalk which allows the tumor to prolapse into the left ventricle during diastole. However, a definitive transthoracic echocardiographic diagnosis of a left atrial myxoma is difficult when the mass does not prolapse, the site of origin cannot be identified. We report a patient with multiple recurrent cerebral infarction and central retinal artery occlusion, determined to have a left atrial mass simulating a thrombus by transthoracic echocardiography at first. A left atrial mass delayed diagnosed as atrial myxoma and resulted in left eye’s vision loss although the myxoma was resected.

## Case report

A 55-year-old woman who presented to the emergency department reported right sided hemiparesis, sudden vision loss in her left eye. She had history of hypertension. Neurologic examination revealed muscle weakness of the right upper and lower limbs. She was found with visual acuities of 20/25 OD and light perception OS. Intraocular pressure was 16 mmHg OD and 16 mmHg OS. Fundus examination of the left eye showed whitening and opacification of the retina with a cherry-red spot in the fovea. Her electrocardiogram showed a normal sinus rhythm. Laboratory findings showed no abnormalities.

**Fig. 1:**
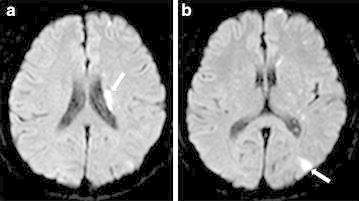
Brain diffusion-weighted magnetic resonance imaging. **a** Multiple embolic infarction in left basal ganglia are seen (*arrow*). **b** In parietotemporal lobe (*arrow*)

**Fig. 2:**
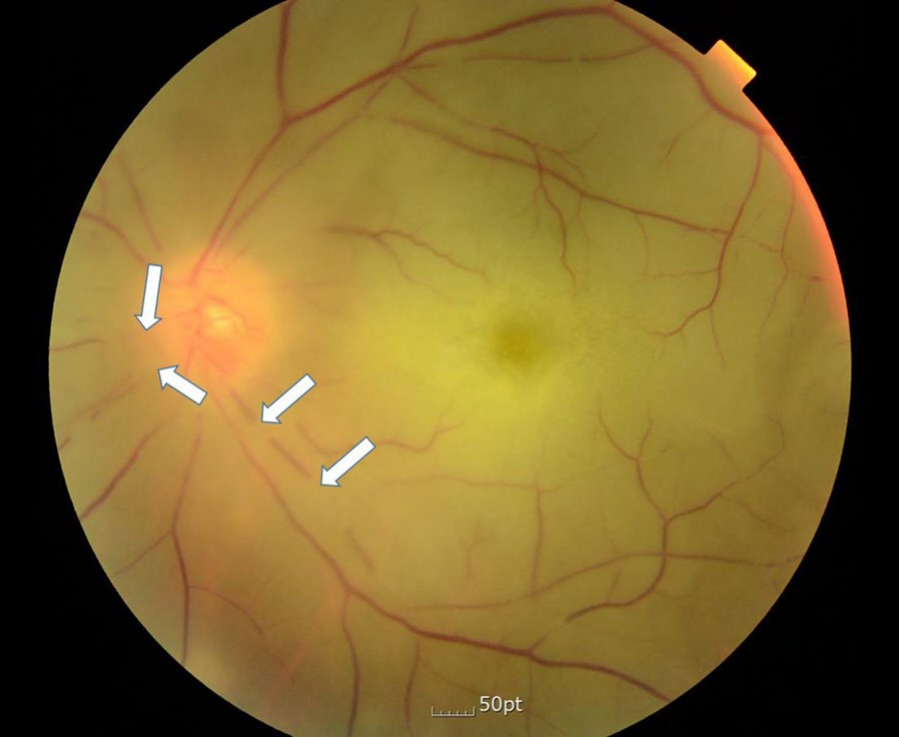
Color fundus photography of the left eye: central retinal artery occlusion; retinal artery attenuation (*arrows*) and optic disc pallor

**Fig. 3:**
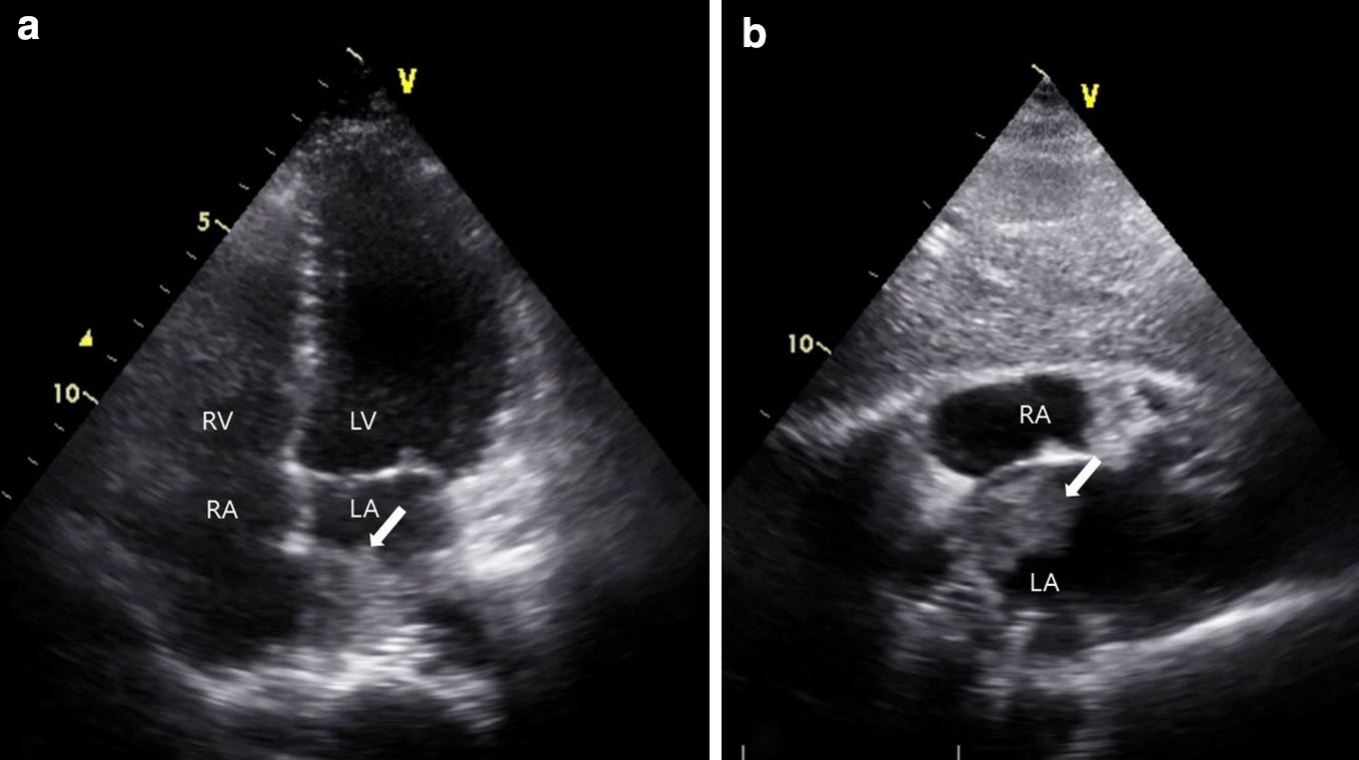
Transthoracic echocardiographic findings. **a** Mass with irregular margin in the left atrium and unclear site of attachment (in *four chamber view*), **b** (in *subcostal view*). *LA* left atrium, *RA* right atrium, *LV* left ventricle, *RV* right ventricle

Diffusion weighted magnetic resonance imaging (MRI) demonstrated multiple hyperintense areas in the left basal ganglia, parietotemporal lobe, suggestive of acute embolic infarction (Fig. 1a, b). Fundus examination of the left eye showed that multiple branches of the retinal arteries were occluded (Fig. 2, indicated as arrows). Transthoracic eocardiography revealed a left atrial mass of the diameter 3 × 3 cm containing a slightly mobile mass and unclear site of attachment (Fig. 3a, b). Transthoracic echocardiography exhibited a large mass (3 × 3 cm sized) with wide base, located at right wall of the high left atrium in the vicinity of right upper and lower pulmonary vein.

**Fig. 4:**
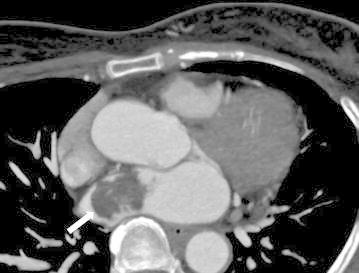
Coronary computed tomography angiogram. Large mass is located in the left atrium and appears heterogeneously low attenuating. The point of attachment appears to be the interatrial septum

At first we thought this mass as thrombus causing multiple emboli. So she was treated with the unfractionated heparin and we planned to recheck the size of mass in the left atrium after 7 days. Five days since admitted, she suddenly fell down and complained aggrevated weakness of the right limbs. We rechecked transthoracic echocardiography and coronary computed tomography angiogram (Fig. 4). Followed transthoracic echocardiography demonstrated no interval change regarding size of mass, further coronary computed tomography angiogram showed the mass had broad stalk from the atrial septum. Therefore, we diagnosed the patient with multiple, recurrent cerebral embolism and retinal artery occlusion due to a left atrial myxoma.

**Fig. 5:**
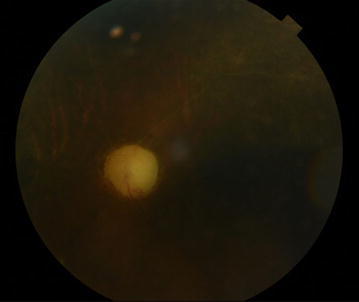
Followed color fundus photography of the left eye: multiple fibrous changes on retina and choroid

To prevent recurrence, we decided to resect the mass and immediately transferred her to a tertiary hospital. She underwent the resection of the myxoma in a teritary hospital. Microscopic analysis of surgical specimens confirmed that it was the atrial myxoma attached to the atrial septum. The patient uneventfully recovered from cardiac surgery, but unfortunately her left eyesight did not come back. Followed fundus examination of the left eye demonstrated optic disc atrophy and chronic ischemic signs (Fig. 5).

Written informed consent was obtained from the patient for publication of this case report and any accompanying images (IRB File No. 0749-160120-HR-022-01).

## Discussion

Retinal artery occlusion caused by acute ischemia of the retina. It may lead to severe irreversible visual impairment. This condition mostly occurs in patients with high blood pressure, heart disease, diabetes or carotid atherosclerosis in elderly people (Varma et al. 2013). Central retinal artery occlusion (CRAO) can be divided into four different subclasses; (1) Non-arteritic permanenet CRAO (2) Non- arteritic transietn CRAO (3) Non-arteriti CRAO with cilioretinal sparing (4) Arteritic CRAO. The majority of CRAOs are caused by platelet fibrin thrombi and emboli as a result of atherosclerotic disease and account for over two-third of all CRAO cases (Chen and Lee 2008).

A cardiac myxoma seems to be a rare cause of vascular problem in the eye, however, ocular episodes due to emboli of a cardiac tumor have been observed in the literature (Schmidt et al. 2005). Physicians should also be alert that myxoma may cause multiple embolic problems, including central retinal arteries occlusion. But we misdiagnosed the cardiac mass as thrombus through transthoracic echocardiography because of unusual attached site and morphology. Mostly cardiac myxoma is attached by a pedicle to the fossa ovalis in the atrial septum but in this case the attached site of the mass get confused at first.

On the contrary to this case, thrombus mimicking cardiac tumors in the atrium have been published (Kmetzo et al. 1985). It means difficult to differentiate between tumors and thrombus. In cases like the present where there is no high risk factor for cerebrovascular disorders or atrial fibrillation, it is important to consider the possibility of myxoma as a cause of multiple embolism despite its rarity. And it demonstrates the utility of transesophageal echocardiography or computed tomography angiogram in distinguishing myxoma from thrombus, if there is not typical features in transthoracic echocardiography (Obeid et al. 1989; Grebenc et al. 2002). Yu et al. reported a 43 year old woman diagnosed with retinal artery occlusion caused by atrial myxoma (Yu et al. 2014). Exact and rapid treatment of atrial myxoma improved patient’s visual capacity. But in our case, we diagnosed the atrial myxoma lately and clinical result was worse.

## Conclusions

We report this case of multiple embolism including central retinal artery occlusion caused by left atrial myxoma that mistook as the cardiac thrombus. Physicians should consider the possibility of myxoma in patient with retinal occlusion and timely management will be beneficial for better outcomes and prognosis. In addition, our report will let ophthalmologists know that cardiac myxoma can cause retinal artery occlusion and proper treatment for myxoma is very important for visual capacity.
